# Malaria Prevention during Pregnancy—Is There a Next Step Forward?

**DOI:** 10.1371/journal.pmed.1001734

**Published:** 2014-09-23

**Authors:** Richard W. Steketee

**Affiliations:** Malaria Control and Elimination Program at PATH, Seattle, Washington, United States of America; PLOS Medicine, United Kingdom

## Abstract

Richard Steketee discusses the two studies by Clara Menendez and colleagues that describe using mefloquine for the intermittent preventative therapy of malaria in both HIV positive and HIV negative pregnant women and outlines the next steps.

*Please see later in the article for the Editors' Summary*

**Linked Research Articles**

This Perspective discusses the following new studies published in *PLOS Medicine*:

González R, Mombo-Ngoma G, Ouédraogo S, Kakolwa MA, Abdulla S, et al. (2014) Intermittent Preventive Treatment of Malaria in Pregnancy with Mefloquine in HIV-Negative Women: A Multicentre Randomized Controlled Trial. PLoS Med 11(9): e1001733. doi:10.1371/journal.pmed.1001733

Clara Menéndez and colleagues conducted an open-label randomized controlled trial in HIV negative pregnant women in Benin, Gabon, Mozambique, and Tanzania to evaluate the safety and efficacy of mefloquine compared to sulfadoxine-pyrimethamine for intermittent preventative therapy for malaria.

González R, Desai M, Macete E, Ouma P, Kakolwa MA, et al. (2014) Intermittent Preventive Treatment of Malaria in Pregnancy with Mefloquine in HIV-Infected Women Receiving Cotrimoxazole Prophylaxis: A Multicenter Randomized Placebo-Controlled Trial. PLoS Med 11(9): e1001735. doi:10.1371/journal.pmed.1001735

Clara Menéndez and colleagues conducted a randomized controlled trial among HIV positive pregnant women in Kenya, Mozambique, and Tanzania to investigate the safety and efficacy of mefloquine as intermittent preventative therapy for malaria in women receiving cotrimoxazole prophylaxis and long-lasting insecticide treated nets.

The malaria parasite lives most of its life and does the majority of its replication in red blood cells. The biologically and immunologically unique utero-placental blood space (where the mother's body must recognize the fetus as not-self and then accept and nurture this foreign body) serves as a near-perfect protected resting and reproducing place for malaria-infected red blood cells–a “holiday spa for parasites.” For a young woman who has grown up with endemic malaria, this protected parasite replication can overcome her acquired systemic malaria immunity and still cause her and her fetus harm in the form of maternal illness, anemia, and premature and low birth weight delivery. For never-exposed young women, the unchecked parasite replication can be catastrophic, leading to severe maternal illness and possible fetal death. Thus, there is no safety from malaria during pregnancy, across the spectrum from very high to very low transmission settings.

This burden of malaria during pregnancy was first described in the scientific literature in 1935 [1] and further elucidated through studies in the 1950s to 1980s [2]; the specific additional burden in HIV-infected women was described in the 1990s as the HIV epidemic spread widely [3],[4]. Interventions initially focused on personal protection and, specifically, antimalarial drug prophylaxis [5]. By 1986, the observed high rates of infection at first antenatal care clinic visit led to a recommendation of an initial antimalarial treatment dose to clear those infections, followed by regular prophylaxis [6]; and most of the emphasis was on the use of chloroquine (CQ) as a safe drug, albeit in the midst of evolving parasite resistance to CQ and growing recognition that adherence to weekly CQ prophylaxis was poor and often less than 20% [7],[8]. A number of studies in the subsequent years elucidated the value of intermittent preventive treatment in pregnancy (IPTp) using an effective curative dose that also provided prophylaxis and could be given as directly observed therapy (DOT) at an antenatal care clinic. The current WHO recommendation includes IPTp along with good preventive strategies (e.g., consistent use of insecticide-treated mosquito nets) and prompt clinical management of malaria illness using diagnosis and antimalarial treatment [9]. Sulfadoxine-pyrimethamine (SP) became an obvious IPTp alternative after CQ as it was curative, had good prophylaxis duration and was a well-tolerated single-dose treatment that could easily be given as DOT [10].

Unfortunately, antimicrobial drug efficacy never lasts. As with CQ, evolving SP resistance was just a matter of time and in the last decade has emerged across much of Africa [11]. Investigators began testing the next options, and as Menéndez and colleagues describe in this issue of *PLOS Medicine* [12],[13], it is not easy to find the next safe and well-tolerated, single-dose drug that elicits prompt parasite clearance and prophylaxis benefit for suitable duration and that can be given as DOT. Their effort to explore whether a low treatment dose (15 mg/kg) of mefloquine (MQ) would be suitable as an alternative to SP for IPTp in HIV-negative women or as IPTp in combination with daily cotrimoxazole in HIV-positive women, showed that reduced rates of malaria infection and maternal illness could be obtained by this efficacious drug, but no obvious added benefit for fetal maturation and growth could be shown for either HIV-negative or HIV-positive women. And, MQ was simply not well tolerated: nausea and dizziness were frequent and potentially debilitating. Previous concerns about the possible association between MQ and fetal loss [14] seem to have been allayed by this study, but the limited added benefit and the poor tolerance still keeps it from being recommended. This report on MQ and its failure to become a viable alternative to SP for IPTp is not alone. Late last year, Pfizer stopped supporting trials of CQ+azithromycin for IPTp (personal communications: George Jagoe, Medicines for Malaria Venture, July 22, 2014) because early results suggested no additional benefit compared to IPTp with SP. As Menéndez and colleagues note, the current WHO recommendations for IPTp with SP remain unchanged.

Luckily, IPTp with SP still demonstrates substantial value in the presence of documented SP-resistance and growing frequency of mutant markers signifying resistance [15],[16]. More importantly, malaria control in many settings has markedly improved with the effect of driving down transmission and infection rates across populations, including among reproductive-age and pregnant women. This multi-site study by Menéndez and colleagues included a number of countries where malaria infection rates in pregnant women are now one-half to one-quarter the levels of just 5–10 years ago. With improving control and growing emphasis on malaria transmission reduction and elimination, this reduced infection burden presents an opportunity to re-examine the strategies for prevention in pregnancy across the wide malaria transmission spectrum.

Any future strategy for malaria prevention in pregnancy must first recognize that for biologic and immunologic reasons, pregnant women and their fetuses and newborns will remain a high-risk population across all transmission intensities; thus, attention to this group must persist. Second, improved malaria control and serious transmission reduction leading to elimination will benefit all, especially reproductive-aged women entering a pregnancy malaria-free and then staying malaria-free for the entire pregnancy. Third, the value of the tradition of women using antenatal care services must be reinforced, and as part of that reinforcement, malaria prevention and case management should be strengthened as a proven service provided in areas where malaria remains a risk. Where malaria risk is high, IPTp should be part of antenatal care service, as currently recommended. Where the malaria risk is much lower (because of historically low transmission intensity or more recent changes with improved control), intermittent screening using prompt diagnosis by rapid diagnostic tests and treatment of infected women (known as intermittent screening and treatment in pregnancy, or ISTp) [17] with appropriate effective antimalarial therapy may become an increasingly important approach, especially if diagnostic tests can be improved and shown to be highly sensitive in detecting infection.

Several other trials are ongoing within and outside of Africa, evaluating potential alternatives to IPTp including new (notably piperaquine-based combinations) and old (chloroquine) long-acting drugs and evaluating ISTp using dihydroartemisinin-piperaquine or artemether-lumefantrine (http://www.mip-consortium.org/). Results are eagerly anticipated later this year and next. Of the truly new drugs still in discovery [18], some may have highly desirable properties in terms of curative and prevention dosing, but experience with use during pregnancy will likely be a long time coming. If intermittent testing is considered central to treatment decision making, improved rapid diagnostic tests will be needed.

As with many well-done clinical trials, the research presented by Menéndez and colleagues does not offer an immediate improvement to malaria prevention in pregnancy. However, it helps clarify that progress has been made in reducing the malaria burden in pregnancy, and by likely closing a door on IPTp with mefloquine, it opens other doors for further important work in the coming years.
